# Kinetic Characterization of 100 Glycoside Hydrolase Mutants Enables the Discovery of Structural Features Correlated with Kinetic Constants

**DOI:** 10.1371/journal.pone.0147596

**Published:** 2016-01-27

**Authors:** Dylan Alexander Carlin, Ryan W. Caster, Xiaokang Wang, Stephanie A. Betzenderfer, Claire X. Chen, Veasna M. Duong, Carolina V. Ryklansky, Alp Alpekin, Nathan Beaumont, Harshul Kapoor, Nicole Kim, Hosna Mohabbot, Boyu Pang, Rachel Teel, Lillian Whithaus, Ilias Tagkopoulos, Justin B. Siegel

**Affiliations:** 1 Biophysics Graduate Group, University of California Davis, California, United States of America; 2 Genome Center, University of California Davis, Davis, California, United States of America; 3 Department of Chemistry, University of California Davis, Davis, California, United States of America; 4 Department of Biochemistry & Molecular Medicine, University of California Davis, Davis, California, United States of America; 5 Department of Biomedical Engineering, University of California Davis, Davis, California, United States of America; 6 Department of Computer Science, University of California Davis, Davis, California, United States of America; King's College London, UNITED KINGDOM

## Abstract

The use of computational modeling algorithms to guide the design of novel enzyme catalysts is a rapidly growing field. Force-field based methods have now been used to engineer both enzyme specificity and activity. However, the proportion of designed mutants with the intended function is often less than ten percent. One potential reason for this is that current force-field based approaches are trained on indirect measures of function rather than direct correlation to experimentally-determined functional effects of mutations. We hypothesize that this is partially due to the lack of data sets for which a large panel of enzyme variants has been produced, purified, and kinetically characterized. Here we report the *k*_cat_ and K_M_ values of 100 purified mutants of a glycoside hydrolase enzyme. We demonstrate the utility of this data set by using machine learning to train a new algorithm that enables prediction of each kinetic parameter based on readily-modeled structural features. The generated dataset and analyses carried out in this study not only provide insight into how this enzyme functions, they also provide a clear path forward for the improvement of computational enzyme redesign algorithms.

## Introduction

The ability to rationally reengineer enzyme function using computational approaches has the potential to enable rapid development of highly efficient and specific catalysts tailored for needs beyond those selected for during natural evolution. [1] A growing route for engineering enzyme catalysts is the use of computational tools to evaluate potential mutations *in silico* prior to experimental characterization. Using the Rosetta Molecular Modeling Suite, reengineering of both specificity and chemistry has been accomplished. [2,3,4,5,6] However, often less than ten percent of designs engineered using this force-field based approach are found to have the intended functional effect. Furthermore, there have been no reports evaluating the predictive power of the Rosetta Molecular Modeling Suite on the functional effects of enzyme mutations. Therefore efforts to both evaluate and improve the predictive power of this computationally inexpensive and widely accessible algorithm are necessary.

The use of large datasets to train and evaluate force-field based algorithms for protein function has been previously validated in the context of protein thermostability. For example, the ProTherm database has over twenty thousand measured effects of mutations on thermostability, and serves as the gold standard for the development of numerous algorithms developed to predict effects of mutations on thermostability. [7,8,9] Current algorithms for protein redesign, in contrast, are not directly trained on experimentally measured effects, but rather indirect measures such as sequence recovery (*i*.*e*. the ability to recapitulate a known active site after running a design simulation). While there have been several previous efforts to construct large families of functionally-characterized mutants, none have produced, purified, and measured the kinetic constants of more than twenty mutants. [10,11,12,13,14] In order to develop algorithms for the rational modulation of kinetic parameters, we hypothesize that it will be necessary to develop libraries of mutant enzymes for which the functional effects of mutations on catalytic efficiency (*k*_cat_/K_M_), apparent substrate affinity (estimated by K_M_), and turnover rate (*k*_cat_) have been measured.

**Fig 1:**
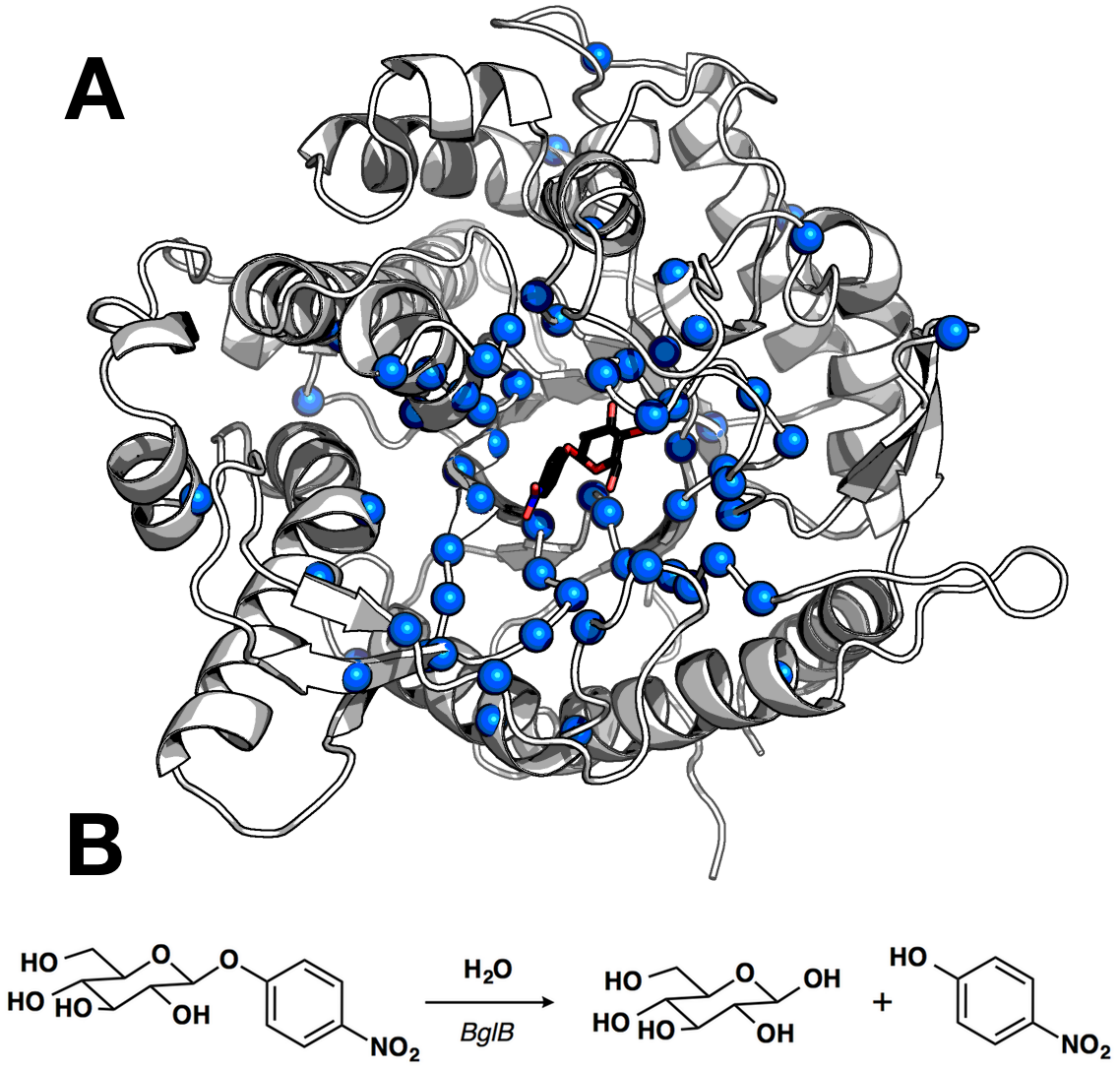
Structure and catalyzed reaction of BglB. (A) Structure of BglB in complex with the modeled *p*-nitrophenyl-β-D-glucoside (pNPG) used for design. Alpha carbons of residues mutated shown as blue spheres. The image was drawn with PyMOL. [16] (B) The BglB–catalyzed reaction on pNPG used to evaluate kinetic constants of designed mutants.

Here, we take the first step towards developing a data set of enzyme mutants with measured effects on kinetic constants that is both large enough and has a wide enough dynamic range to enable training of computational protein design algorithms. The initial enzyme of focus is a family 1 glycoside hydrolase: β-glucosidase B (BglB) from *Paenibacillus polymyxa*. The family 1 glycoside hydrolases have been the subject of numerous structural and kinetic studies due to their importance as the penultimate step in cellular ligno-cellulose utilization. [15] The structure of BglB indicates that it follows a classical Koshland double-displacement mechanism in which E353 performs a nucleophilic attack on the anomeric carbon of the substrate’s glucose moiety. The leaving group is protonated by E164. A third active site residue, Y295, orients E353 for catalysis with a hydrogen bond. [15] The protein structure and reaction scheme are provided in Fig 1.

In this study we report the largest data set of its kind, in which 100 mutants of BglB are produced, purified, and kinetically characterized (*i*.*e*., kinetic constants *k*_cat_, K_M_, and K_i_ measured) using the reporter substrate *p*-nitrophenyl-β-D-glucoside (pNPG). The production of this dataset revealed several mutations to non-catalytic residues (*i*.*e*. those not directly involved in the proposed reaction chemistry) that are as important to the enzyme-catalyzed reaction as catalytic residues. In addition, we demonstrate the ability to use this dataset to train computational algorithms for the prediction of *k*_cat_, K_M_, and *k*_cat_/K_M_ using readily-calculated metrics derived from molecular modeling. Finally, we illustrate how machine learning can be used to identify structural features from the molecular models that significantly improve the predictive accuracy of the molecular modeling. These analyses provide insight into the factors important for catalysis in BglB as well as a path forward for the development and evaluation of next-generation enzyme reengineering algorithms.

## Results

### Computationally-directed engineering of BglB

A crystal structure (PDB 2JIE) of recombinant BglB in complex with the substrate analog 2-deoxy-2-fluoro-α-D-glucopyranose was used to identify the substrate binding pocket and the catalytic residues. To generate a molecular model representative of a proposed transition state in the hydrolysis of pNPG, an S_N_2-like transition state structure was built and minimized in Spartan based on a 3D conformer of PubChem CID 92930. Functional constraints were used to define catalytic distances, angles, and dihedrals between pNPG, the acid-base E164, the nucleophile E353, and Y295, which is proposed to orient the nucleophilic glutamate. The angle between the attacking oxygen from E353, the anomeric carbon, and the phenolic oxygen was constrained to 180°, in accordance with an S_N_2-like mechanism. [17] A complete set of files that were used for modeling are provided in S1 Code.

Two approaches were used to establish a set of mutants to generate and kinetically characterize. The first approach was a systematic alanine scan of the BglB active site where each residue within 12 Å of the ligand in our model was individually mutated to alanine. In the second approach, mutations predicted to be compatible with the modeled pNPG transition state in BglB structure were selected by students learning about molecular modeling through the program Foldit, a graphical user interface to the Rosetta Molecular Modeling Suite. [4,18] Mutations were modeled and scored in Foldit and a selection of mutations that were either favorable or did not increase the energy of the overall system by greater than 5 Rosetta energy units were chosen to synthesize and experimentally characterize. Fig 1A illustrates the positions in the protein where mutations were introduced, and the complete set of mutations selected is listed in S1 Table. A total of 69 positions were covered over the 100 mutants made.

### Protein production and purification

Each of the 100 mutants was made via Kunkel mutagenesis [19] using the Transcriptic cloud laboratory platform and verified by Sanger sequencing. Plasmids containing the mutant genes were transformed into *Escherichia coli* BL21(DE3), 5 mL cultures grown in Terrific Broth and expression induced with IPTG. Proteins were purified via immobilized metal affinity chromatography and eluted in 200 μL HEPES buffer, as described in detail in S1 Text. The absorbance at 280 nm of eluted protein was used to quantify protein yield and SDS-PAGE was used to evaluate purity (S1 Fig). All proteins used in this study were greater than 80% pure, and fresh resin was used for each mutant to prevent wild type contamination.

**Fig 2:**
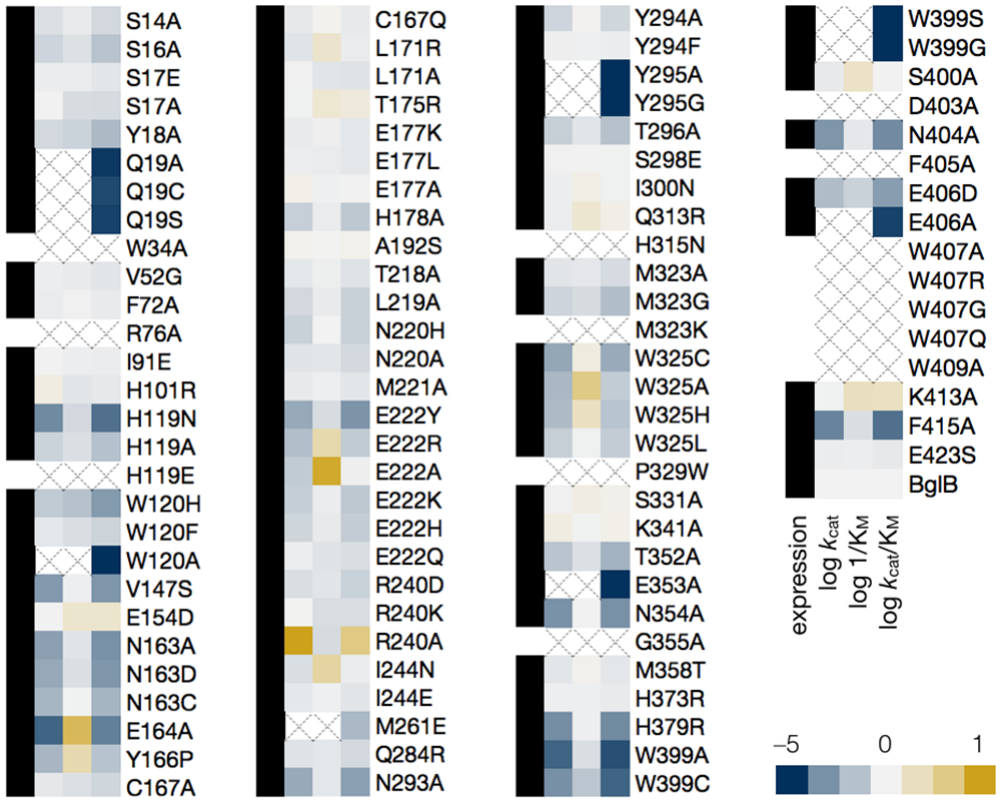
Log scale relative kinetic constants of 100 BglB mutants. The heatmap depicts the effect of each mutation on each kinetic constant relative to native BglB, normalized at 0. As indicated in the color legend, gold is for higher value and blue for a lower value. The metric 1/K_M_ is used so a higher value is consistently corresponding to a “better” kinetic constant (assuming a lower K_M_ is better) when evaluating *k*_cat_, *k*_cat_/K_M_, and K_M_. If the kinetic constant was not measurable, an X is depicted in the box. Proteins that were expressed as soluble protein with a final purification concentration of >0.1 mg/mL and validated by SDS-PAGE are labeled with a black box in the first column. Those below our limit of detection of 0.1 mg/mL are labeled with an empty box. Values are on a log scale and the ranges are as follows: 10–11,000 min^-1^ (*k*_cat_), 0.6–85 mM (K_M_), and 10–560,000 M^-1^min^-1^ (*k*_cat_/K_M_) with wild type constants of 880 ± 10 min^-1^, 5.0 ± 0.2 mM, and 171,000 ± 8000 M^-1^ min^-1^ for *k*_cat_, K_M_, and *k*_cat_/K_M_ respectively. A full table of kinetic constants and substrate versus velocity curves for each are provided in S1 Table and S3 Fig.

A total of ten biological replicates of the native BglB were used to assess expression and purification. The average concentration of proteins after purification was found to be 1.2 ± 0.4 mg/mL. Of the 100 mutants synthesized, 89 express and purify as soluble protein (Fig 2). The final concentrations for all 100 mutants are included in S1 Table. Greater than 35% maintained the yields obtained for native BglB, and 15% did not express and purify as a soluble protein above our limit of detection (0.1 mg/mL) for protein yield after purification based on A_280_ and SDS-PAGE.

### Kinetic characterization of mutants

Michaelis-Menten kinetic constants for each of the 100 mutants were determined using the colorimetric assay of pNPG hydrolysis. The results are represented as a heatmap in Fig 2. Ten biological replicates of the wild type enzyme have an average *k*_cat_ of 880 ± 10 min^–1^, K_M_ of 5.0 ± 0.2 mM, and *k*_cat_/K_M_ of 171,000 ± 8,000 M^–1^ min^–1^. To determine kinetic constants, observed rates at 8 substrate concentrations were fit to the Michaelis-Menten equation. If no clear saturation was observed then a linear equation was used to determine *k*_cat_/K_M_. Experimentally measured kinetic constants and nonlinear regression analysis for each mutant can be found in S1 Table and S3 Fig, respectively.

Based on the maximum concentration of enzyme used in our assays and colorimetric absorbance changes at the highest substrate concentration used, we estimate our limit of detection for *k*_cat_/K_M_ to be 10 M^-1^min^-1^. Of the 89 solubly purified mutants, 6 are below the limit of detection. The highest catalytic efficiency observed is 560,000 M^-1^min^-1^ for mutation R240A. In addition, while no substrate inhibition is observed for the wild type BglB, four mutants exhibit measurable substrate inhibition (the inhibition parameter K_i_ for only these mutants is reported in S1 Table as it was not measurable for most mutants).

### Observed sequence–structure–function relationships in BglB

In agreement with previous studies, our results demonstrate the importance of E164, E353, and Y295 for catalysis. Mutating any of these residues to alanine results in a >85,000-fold reduction in catalytic efficiency (*k*_cat_/K_M_). However, beyond the catalytic residues, the systematic alanine scan of every residue within 12 Å of the ligand revealed mutations which have an equivalent functional effect to mutating the established catalytic residues to alanine.

**Fig 3:**
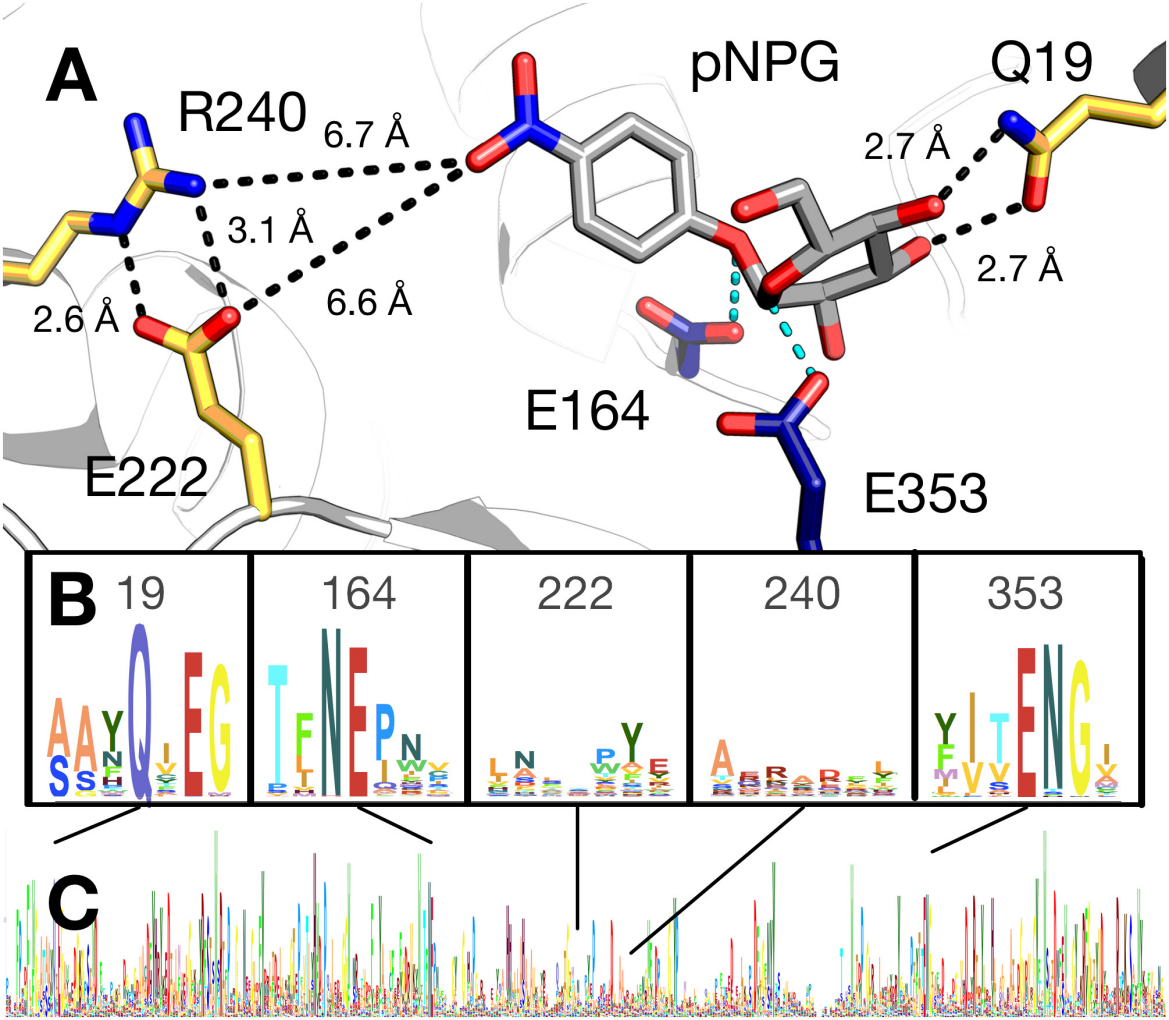
Active site model and conservation analysis of BglB. (A) Docked model of pNPG in the active site of BglB showing established catalytic residues (navy) and a selection of residues mutated (gold). A multiple sequence alignment of the Pfam database’s collection of 1,554 family 1 glycoside hydrolases was made and the sequence logo for (B) selected regions around specific residues discussed in the text and (C) over the entire BglB coding sequence is represented. The height for each amino acid indicates the sequence conservation at that position.

For example, the Q19A mutant showed a dramatic effect on function: catalytic efficiency decreased by 57,000-fold. Analysis of the crystal structure of BglB suggests that both the nitrogen and oxygen of the amide sidechain interact with hydroxyl groups on the substrate (Fig 3A). A multiple sequence alignment of the BglB enzyme family in the Pfam database (including 1,554 non-redundant proteins), revealed that Q19 is 95% conserved in this family (Fig 3B). Unlike E353, the nucleophilic glutamate directly involved in the reaction chemistry, Q19 is not directly involved in the reaction. This is consistent with the theory that orientation of the substrate is a critical aspect of catalysis ("orbital steering") for which Q19 is likely crucial. [20] A crystal structure of BglB Q19A in complex with the 2-deoxy-2-fluoro-α-D-glucopyranose inhibitor may help elucidate the structural effect of this mutation. Based on molecular modeling, no major structural change for this mutant is predicted (S2 Fig).

A novel finding was a tenfold increase of *k*_cat_ by a single point mutant, R240A. The BglB crystal structure reveals that R240 forms two hydrogen bonds with E222 (Fig 3A). Molecular modeling of the R240A mutant predicts that E222 would adopt an alternative conformation in which the acid functional group of the glutamate is 2 Å closer to the active site (S2 Fig). This would likely result in a significant change of the electrostatic environment around the active site, and indicates that the electronegative environment enhances catalysis of pNPG hydrolysis. Consistent with this hypothesis is the observation that the mutation E222A decreases *k*_cat_ by tenfold. Both observations support previous evidence that the electrostatic environment of an enzyme's active site is of primary importance to catalysis. [21]

### Conservation analysis of the BglB active site

Of the 44 positions in the active site systematically mutated to alanine, 11 are conserved by >85% in amino acid identity with respect to 1,554 homologues in the Pfam database. When any one of these amino acids is mutated to alanine, catalytic efficiency decreases >100-fold (S3 Table). This supports the widely held assumption that highly conserved residues within an enzyme active site are functionally important. However, only 11 of the 44 residues within 12 Å of the active site are >85% conserved. Of the 33 remaining residues within 12 Å of the active site, only 8 alanine mutations resulted in a decrease in catalytic efficiency of greater than 100-fold, and 10 of these 33 mutations were not found to significantly affect catalytic efficiency.

Based on these findings, there does not appear to be a strong correlation between residue identity and function if a particular residue is <85% conserved. This observation supports the hypothesis that native sequence recovery is not a good metric for training design algorithms. In addition, the mutation R240A, which is not observed in any natural variant in the glycoside hydrolase 1 family, resulted in a 10-fold increase in *k*_cat_ on pNPG. This emphasizes the importance of not limiting design efforts to changes previously observed in nature when engineering function towards a new substrate.

### Computational modeling and evaluation of predictive ability

In order to evaluate the Rosetta Molecular Modeling Suite’s ability to predict the functional effects of mutations on BglB kinetic properties, molecular models were generated for each of the 100 BglB mutants. For each mutant, the modeled pNPG previously described was docked into the active site by a Monte Carlo simulation with random perturbation of the ligand followed by functional constraint optimization through rigid body minimization of the ligand, sidechain and ligand conformational sampling, and, finally, ligand, sidechain, and backbone minimization. This protocol was used to mimic protocols used in successful enzyme reengineering efforts. [2] An example set of input files for wild type BglB are provided in S1 Code.

For each mutant, 100 models were generated as described above and the lowest 10 in overall system energy for each mutant were selected for subsequent structural analysis. A value for each of 59 potentially informative features (such as predicted interface energy, number of hydrogen bonds between protein and ligand, and change in solvent accessible surface area upon ligand binding) was calculated for each model. Correlation of the average calculated structural features to each kinetic constant was assessed using Pearson Correlation Coefficient (PCC) and Spearman Rank Correlation (SRC). For both *k*_cat_/K_M_ and *k*_cat_, the strongest correlation observed is to the total number of non-local contacts (count of residues separated by more than 8 sequence positions that interact with each other), with a PCC of 0.57 (p-value 0.009; Wilcoxon test) and 0.43 (p-value 0.004; Wilcoxon test), respectively. For 1/K_M_, the highest PCC is 0.29 (p-value 0.0005; Wilcoxon test) to the total number of hydrogen bonds in each BglB model. The SRC follows similar trends to PCC for all three predicted constants (SRC of 0.55, 0.42 and 0.38 for *k*_cat_/K_M_, *k*_cat_ and 1/K_M_ respectively). The PCC and SRC values for all features are available in S2 Table.

### Machine learning prediction of kinetic constants

Because no single structural feature predicts *k*_cat_, 1/K_M_, or *k*_cat_/K_M_ with high accuracy, machine learning techniques were used to identify a subset of calculated features correlated to observed kinetic constants. Elastic net regularization, a constraint regression technique that uses both l_1_ and l_2_ regularization for feature selection, was used to identify structural features that could be combined in order to predict each kinetic constant. To remove bias, we used an ensemble learning technique, where the predicted value was an average of 1000 elastic net models, each trained on a different subset of the data.

**Fig 4:**
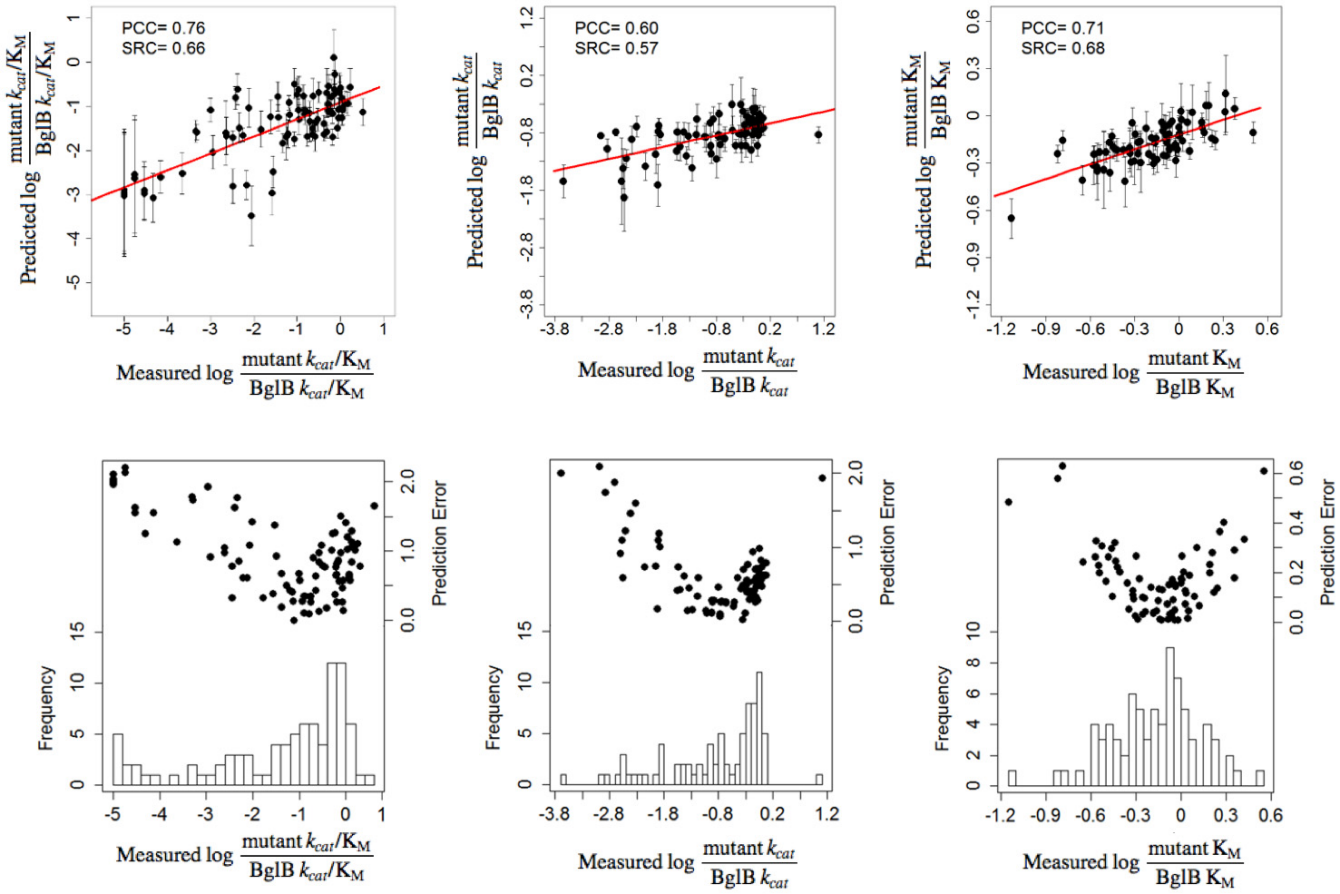
Correlation between machine learning predictions and experimentally-determined kinetic constants. *Top panels*: predicted versus experimentally-measured values for kinetic constants *k*_cat_/K_M_ (A), *k*_cat_ (B), and 1/K_M_ (C). All values are relative to the wild type enzyme and on a log scale. The standard deviation (error bars) of the predicted values are calculated based on the prediction by 1000-fold cross validation for each point. The red line corresponds to linear regression and has been added for visualization purposes. *Bottom panels*: Histograms of experimentally-determined values in the data set (90, 80 and 80 samples for *k*_cat_/K_M_, *k*_cat_, and K_M_, respectively), along with the residual errors (scatter plot) between predicted and measured kinetic values.

The final prediction from this ensemble learning regression method outperformed single feature selection for each kinetic constant. For *k*_cat_/K_M_, the PCC increased to 0.76 from 0.57, in the case of *k*_cat_ to 0.60 from 0.43, and for 1/K_M_ to 0.71 from 0.29. Fig 4 (top panel) illustrates the correlations between machine learning predictions and experimentally-measured values. Fig 4 (bottom panel) depicts the histogram of samples with respect to their measured kinetic constant value and the observed error between predicted and measured value.

The primary features found to correlate to 1/K_M_ are metrics of protein packing without the ligand present (*i*.*e*. a minimal number of voids). All of these packing features are positively correlated to 1/K_M_, meaning that, in BglB, a decrease in structural packing (*i*.*e*. a higher packing value) around the catalytic residues and protein results in a lower K_M_. A tightly packed enzyme without voids would likely result in pre-ordering of the active site. Therefore this correlation is consistent with BglB requiring a pre-ordered active site for efficient substrate binding, and potentially catalysis. [20] To further support this proposed classical lock-and-key mechanism, the observed root mean square deviation (RMSD) between the crystal structures of the apo (2O9P) and transition state analogue–bound (2JIE) forms of BglB is < 0.2 Å. It is important to note that K_M_ is a complex kinetic constant and not necessarily correlated to substrate binding (*i*.*e*. K_d_). Furthermore, the relationship between K_M_ and K_d_ may change between mutants. However, when modulating enzyme activity K_M_ is a primary parameter of functional interest, and therefore the ability to predictively model K_M_ is of significant importance. Future efforts including mechanistic and structural studies for a diverse set of mutants from this data set will be needed in order to elucidate the detailed molecular mechanism of the discovered relationship between K_M,_ K_d_, and structural packing in BglB.

The features selected by the algorithm as predictive of *k*_cat_ include a count of polar contacts, consistent with mechanistic studies that indicate BglB stabilizes the positive charge on the oxocarbenium ion in the proposed transition state. [22] Another primary featured selected as a predictor of *k*_cat_ by the elastic net algorithm is a ligand burial term (change in solvent accessible surface area on binding) which is consistent with the stabilization of the transition state and catalysis through tight interface packing and shape complementarity. In addition, these features will all have a significant effect on the electrostatic environment of the enzyme active site, and are consistent with effects observed for R240A and E222A on catalysis. However, similar to the analysis of K_M_, the rate-limiting step for BglB under these experimental conditions with this substrate is not known. As the rate limiting step may change between mutants, further studies on the detailed kinetic parameters of the native enzyme and mutants will likely provide further insight into the determinants of function and key structural factors required for high turnover rates.

For BglB, the most informative feature predicting *k*_cat_/K_M_ is the calculated hydrogen bonding energy of the substrate. The identification of this feature by the machine learning algorithm indicates the importance of protein-ligand hydrogen bond interactions. Hydrogen-bonding interactions are exceptionally important for the enzyme-catalyzed reaction, as noted for the Q19A mutation. Strong hydrogen bonding interactions between the protein and substrate are likely of the utmost importance for optimally positioning the substrate and the protein sidechains to enable catalysis ("orbital steering"). [23] This is consistent with the hydrogen bonding energy being selected by machine learning as a feature of primary importance for catalytic efficiency.

While many of the selected features are consistent with well-established mechanisms of enzyme catalysis, there were several unexpected observations. One unexpected trend is that several features are selected as predictive of *k*_cat_/K_M_ but not either *k*_cat_ or K_M_. Further analysis of *k*_cat_ and K_M_ revealed that there is no significant correlation between two parameters in this dataset (S4 Fig). This suggests that *k*_cat_ and K_M_ are independent parameters for BglB, and it is therefore not unexpected that features found to be predictive of *k*_cat_/K_M_ are not predictive of either *k*_cat_ or K_M_ independently.

A second unexpected observation is that the most common metric used for evaluating designs, interface energy, [2,3,4,5,6] is not selected by the algorithm to be predictive of any kinetic constant. Ideally, this would be the single metric optimally correlated with either *k*_cat_ or *k*_cat_/K_M_. This likely stems from training the enzyme design algorithm on indirect measures of function, further supporting the need to train force-field based algorithms on direct experimental measurements.

## Discussion

The Rosetta Molecular Modeling Suite has been successfully used to guide the engineering of a wide range of enzyme functions. However, there has been a limited ability to benchmark its predictive power for enzyme reengineering due to the lack of a large, quantitative dataset correlating the effects of mutations to kinetic parameters over a large dynamic range. Here, we construct the first such dataset and report statistically significant evaluation of our ability to predict the functional effects of enzyme mutations.

The data generated here uncovered new structure-function relationships in BglB, and provides the quantitative contribution towards catalysis of each amino acid in the active site. This systematic analysis revealed that several amino acids in the active site that are not directly involved in the reaction chemistry are almost as important to catalysis as the three residues which are directly involved in the chemistry. This highlights the importance of the entire active site in catalysis. This is consistent with a recent report exploring the interconnectedness of a network of five residues in alkaline phosphatase. [24]

Furthermore, the large dataset of kinetic constants reported here enabled the use of machine learning to select structural features that are predictive of function. It was unexpected to observe that the calculated interface energy is not found to be predictive of any kinetic parameter, and was not a feature selected by machine learning as predictive of function. This has significant implications for future design strategies since interface energy is one of the most common metrics currently used to evaluate redesigned enzyme-ligand interfaces. It may be pertinent to develop additional training datasets, such as we have done for BglB, in order to further quantify the appropriate metrics to be used for selecting designed mutants to functionally characterize in other enzyme systems. Similarly, the development and quantitative characterization of mutant datasets in the case of other enzymes will show which features are general and which are specific to different enzymatic classes.

From the machine learning analysis, an interesting non-linear relationship between predicted and experimental rates is revealed as the residual error increases with the measured kinetic value (Fig 4, bottom panel). There are two factors that contribute to this effect. First, as is evident from the histogram, mutants with lower activity have been sampled more in all cases, and the sampling size per bin tends to bias the error distribution. Second, there are no features or feature combinations in the regression model that correlate well with the observed non-linearity. Non-linear regression methods (second-order polynomial and Poisson kernels) achieved similar performance (data not shown). As such, there is room for improvement in future studies by uniformly sampling the parameter space (which is difficult to predict *a priori* but can be rectified by increasing the sample size). This could be achieved by building on recent high throughput experiments that systematically screen the phenotypic effect of every possible enzyme point mutant. [14] A combination of high throughput screening with molecular modeling could be used to identify a subset of mutants to purify and kinetically characterize in order to maximize the information content when training new algorithms. In addition, introducing informative features that capture different aspects of the variation observed and exploring other non-linear regression methods that balance the bias-variance trade-off could be used to address the non-linear relationship between predicted and experimental kinetic constants.

This work demonstrates how constrained statistical learning can be integrated with measured functional effects of a mutation on enzyme kinetic constants in order to build predictive models. As more datasets of kinetically characterized mutant variants become available for a variety of enzymes, our understanding of how these systems function and our ability to identify the most informative features will increase. Integration of these data-driven methods with enzyme redesign algorithms has the potential to significantly increase the predictive performance of the computational tools that are currently available, with far-reaching applications.

## Conclusion

In this work 100 computationally designed mutants of a family 1 glycoside hydrolase were produced, purified, and kinetically characterized. This dataset revealed new insights into structure-function relationships in BglB. Machine learning protocols were employed to select a subset of readily calculated structural features that are highly predictive of each measured kinetic parameter. The development of this large data set allowed a statistically significant assessment of the Rosetta Molecular Modeling Suite’s ability to predict functional effects of mutations on this enzyme’s kinetic properties. This data set will be invaluable for the development of computational enzyme engineering algorithms and providing insight into the physical basis of enzyme sequence-structure-function relationships.

## Methods

### Molecular modeling for mutant selection

The crystal structure of recombinant BglB in complex with the substrate analog 2-deoxy-2-fluoro-α-D-glucopyranose was used to identify the substrate binding pocket and the catalytic residues. Functional constraints were used to define catalytic distances, angles, and dihedrals among 4-nitrophenyl-β-D-glucoside, E164, E353, and Y295. The structure was then loaded into Foldit, a graphical user interface to Rosetta. Point mutations to the protein were modeled, and a subset were chosen by students learning about molecular modeling. Generally, the designs had energies no more than 5 Rosetta energy units higher than the native structure.

### Mutagenesis, expression, and purification

The BglB gene was codon-optimized for *E*. *coli*, synthesized as a DNA String by Life Technologies, and cloned into a pET29b+ vector using Gibson assembly. [25] Site-directed mutagenesis performed according to the method developed by Kunkel was used to generate mutations to BglB via the Transcriptic cloud laboratory platform. Variants were expressed and purified via immobilized metal ion affinity chromatography and assessed using 4–20% gradient SDS-PAGE Bolt Gels from Life Technologies. More details are provided in S1 Text.

### Kinetic characterization

The activity of the computationally designed enzyme variants was measured by monitoring the production of 4-nitrophenol. Mutant proteins ranging in concentration from 0.1 to 1.7 mg/mL were aliquotted in triplicate in 25 μL volumes and 75 μL of *p*-nitrophenyl-β-D-glucoside (100 mM, 25 mM, 6.25 mM, 1.6 mM, 0.4 mM, 0.1 mM, or 0.02 mM) in enzyme storage buffer was added. Absorbance at 420 nm was measured every minute for 30–60 min and the rate of product production in M/min was calculated using a standard curve (see Supplemental Materials). A total of 2944 observed rates for 119 individual proteins (including biological replicates) were fit to the Michaelis-Menten equation using SciPy.

### Predictive modeling

One hundred molecular models of each mutant enzyme were generated using the Rosetta Molecular Modeling Suite by Monte Carlo optimization of total system energy and the lowest 10 selected for feature generation. Elastic net regularization was used to select the most informative features. We assessed the prediction performance of the method with both 10-fold cross-validation (CV) and bootstrapping. First we performed 10-fold cross-validation (CV) and evaluated the predicted performance on the left-out samples (generalization error) at each of the 10 runs. Then we repeated this procedure (*i*.*e*. the 10-fold CV) 1,000 times to randomize the sample distribution among the folds (Fig 4). That way, we reduce the effect of any bias for evaluating left-out prediction performance. Additionally, we performed bootstrapping by considering sets of size 2n, where n is the number of samples in the whole dataset (90, 80 and 80 samples for *k*_cat_/K_M_, *k*_cat_, and K_M_, respectively). This setting achieves an average coverage of 86.7% of the original data set in any given bootstrapping sample. The left-out samples were then predicted by an elastic net model training on the bootstrapping set. We repeated this procedure 1,000 times and then we averaged the prediction performance of the left-out samples over all runs. As shown in S2 Text, the bootstrapping performance is similar to that of 10-fold CV that is depicted to Fig 4 (slight variations due to smaller training/testing ratio).

The final three feature sets (one of each parameter to be estimated) were selected according to the averaged weight of each feature in all the 10,000 elastic net models (10 models per cross-validation, randomized 1,000 times). The weight of each selected feature in table 1 was normalized with respect to the weight with the largest absolute value. P-values were calculated based on the Wilcoxon signed-rank test after features and kinetic constants were normalized in the [0,1] interval. More information about the optimization and statistical procedure followed is available in S2 Text.

**Table 1:** Most informative structural features predicting each kinetic constant. For each mutant, 10 out of 100 models were selected based on the lowest total system energy. Fifty-nine structural features were calculated for the selected models and the most informative features were selected based on a constrained regularization technique (elastic net with bagging; see Methods). The table contains features that have been assigned non-zero weights during training (9 for *k*_cat_/K_M_, 8 for *k*_cat_, 10 for K_M_). The weights are multiplied by a normalized form of the value (not shown), and can therefore indicate both a positive or negative relationship. For example, a negative weight for hydrogen bonding is consistent with a positive correlation to hydrogen bonding where a smaller number indicates more hydrogen bonding is occurring. Inversely, a positive weight for packing would indicate a positive correlation since a larger value indicates a system with fewer voids. The relative contribution of each feature in determining the kinetic constant is given as a normalized weight (columns 1–3). Column 4 provides a description of each feature, and columns 5 and 6 show the range of observed values in the training dataset. The full feature table is available in S2 Table. *ns = feature not selected by the algorithm*.

*k*_cat_/K_M_	*k*_cat_	1/K_M_	Description	Min.	Max.
-1.00	ns	ns	Hydrogen bonding energy of pNPG	-4.53	-1.8
-0.63	1.00	-0.03	Total number of polar contacts	144	155
-0.43	ns	ns	Count of hydrogen bonds to pNPG	4	9
-0.03	ns	ns	Hydrogen bonding energy of E164	-0.93	-0.21
0.29	ns	-0.27	Lennard-Jones repulsion of Y295	0.54	0.99
0.39	0.92	ns	Change in pNPG solvent-accessible surface upon binding	0.86	0.96
0.44	0.15	1.00	Packing of the system without pNPG	0.67	0.72
0.44	0.53	0.46	Packing of the system with pNPG	0.67	0.73
0.98	0.09	ns	Hydrogen bonding energy of Y295	-1.28	-0.5
ns	-0.51	ns	Packing with pNPG around E353	0.19	1
ns	-0.10	ns	Total system energy	-636.44	-621.6
ns	-0.01	ns	Hydrogen bond energy of the total system	-76.7	-67.63
ns	ns	0.11	Lennard-Jones repulsion around E353	0.67	1.41
ns	ns	0.27	Average hydrophobic surface area without pNPG	0.51	1.75
ns	ns	0.32	Packing around E353 without pNPG	0.37	0.99
ns	ns	0.34	Packing around E164 without pNPG	0.37	0.99
ns	ns	0.38	Packing around Y295 without pNPG	0.34	0.99
ns	ns	0.51	Lennard-Jones repulsion of E164	0.83	1.53

## Supporting Information

S1 Code Rosetta input files. Rosetta input files and associated scripts for generating and scoring *in silico* mutations to the BglB structure.
(ZIP)

S1 Fig SDS-PAGE images for 100 variants of BglB. Gel images showing all proteins used in this study, including replicates of wild type assayed with each batch of mutants. Gels were stained overnight with Coomassie Blue. Protein ladder used was SeeBlue^®^ Plus2 Pre-stained Protein Standard (Life Technologies). Gels were imaged on a BioRad Gel Doc EZ system.
(TIFF)

S2 Fig Active site models of mutants Q19A, R240A, and wild type BglB. The lowest energy of 100 models generated for each mutant is depicted. In panel A, mutation of the glutamine at position 19 to an alanine removes two hydrogen bonds (black) to the substrate compared to wild type (C). In panel B, mutation of the arginine at position 240 to an alanine is predicted to stabilize an alternate conformation of E222A, bringing the carboxylate group to 4.2 Å of the substrate's nitro group. Distances and between the substrate, *p*-nitrophenyl-β-D-glucoside, and the BglB molecule are indicated by black lines.
(TIFF)

S3 Fig Diagnostic plots showing Michaelis-Menten, Michaelis-Menten with substrate inhibition, or linear fit for each of 100 mutants. For each mutant, 8 observed rates (in triplicate) were fit to the Michaelis-Menten equation using SciPy and plots were generated using Matplotlib. Plots were used to visually confirm statistical analysis of the fits.
(PNG)

S4 Fig Plot of the values of log *k*_cat_ versus log 1/K_M_ for 100 mutants relative to wild type BglB, showing the statistical independence of *k*_cat_ and K_M_ in the BglB system. (TIFF)

S1 Table Kinetic constants for 100 computationally-designed BglB mutants. Included are columns (1) the mutation (2) protein yield as assessed by absorbance at 280 nm (3, 4, 5, 6) kinetic constants and nonlinear regression analysis for each of *k*_cat_, K_M_, K_I_, and *k*_cat_ /K_M_.
(DOCX)

S2 Table Correlations between individual structural features and each of *k*_cat_, K_M_, and *k*_cat_/K_M_. PCC and SRC values for each individual structural feature, given by Rosetta short name. For explanation of each short name, see reference [26].
(DOCX)

S3 Table Conservation analysis of BglB active site residues. A multiple sequence alignment of 1,554 family 1 glycoside hydrolases from the Pfam database aligned to the BglB wild type sequence was used for this analysis. Column 1 is the relative *k*_cat_/K_M_ compared to wild type on a log scale. Column two gives the position and native BglB residue at that position. Column three is the percentage of the 1,554 aligned sequences that have the same residue as BglB.
(DOCX)

S1 Text Supplemental materials and methods. (DOCX)

S2 Text Prediction and feature selection via Elastic net. (DOCX)
