# Supplementary figures and images for: Kinetic Characterization of 100 Glycoside Hydrolase Mutants Enables the Discovery of Structural Features Correlated with Kinetic Constants
Source: PLoS One. 2016 Jan 27;11(1):e0147596. doi: 10.1371/journal.pone.0147596 (PMC4729467; doi:10.1371/journal.pone.0147596)

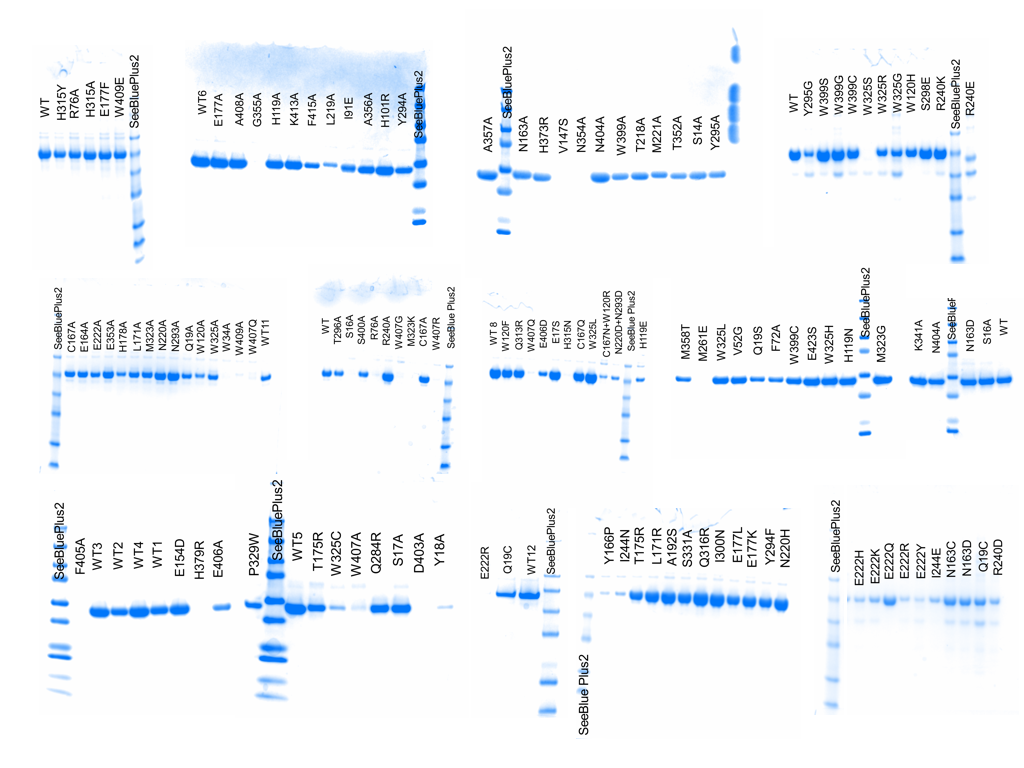

Supplement: S1 Fig — Gel images showing all proteins used in this study, including replicates of wild type assayed with each batch of mutants. Gels were stained overnight with Coomassie Blue. Protein ladder used was SeeBlue® Plus2 Pre-stained Protein Standard (Life Technologies). Gels were imaged on a BioRad Gel Doc EZ system. (TIFF) [file pone.0147596.s002.tiff]

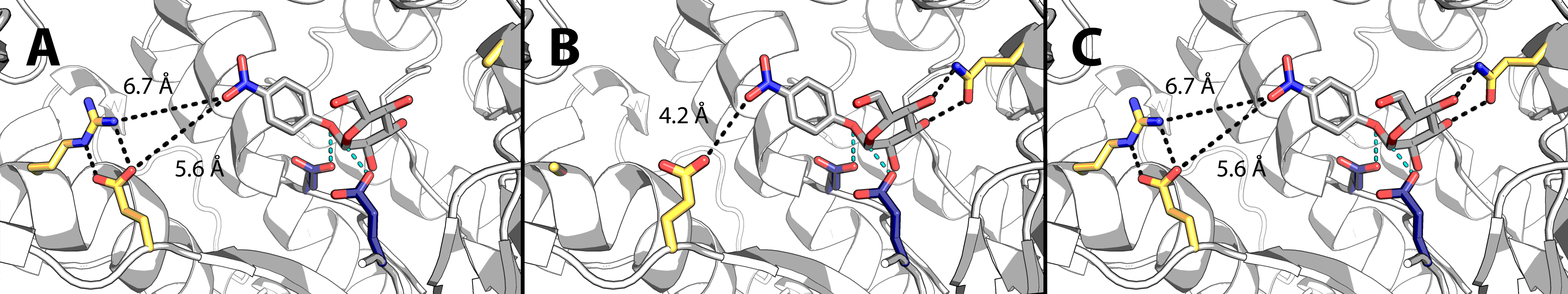

Supplement: S2 Fig — The lowest energy of 100 models generated for each mutant is depicted. In panel A, mutation of the glutamine at position 19 to an alanine removes two hydrogen bonds (black) to the substrate compared to wild type (C). In panel B, mutation of the arginine at position 240 to an alanine is predicted to stabilize an alternate conformation of E222A, bringing the carboxylate group to 4.2 Å of the substrate's nitro group. Distances and between the substrate, p-nitrophenyl-β-D-glucoside, and the BglB molecule are indicated by black lines. (TIFF) [file pone.0147596.s003.tiff]

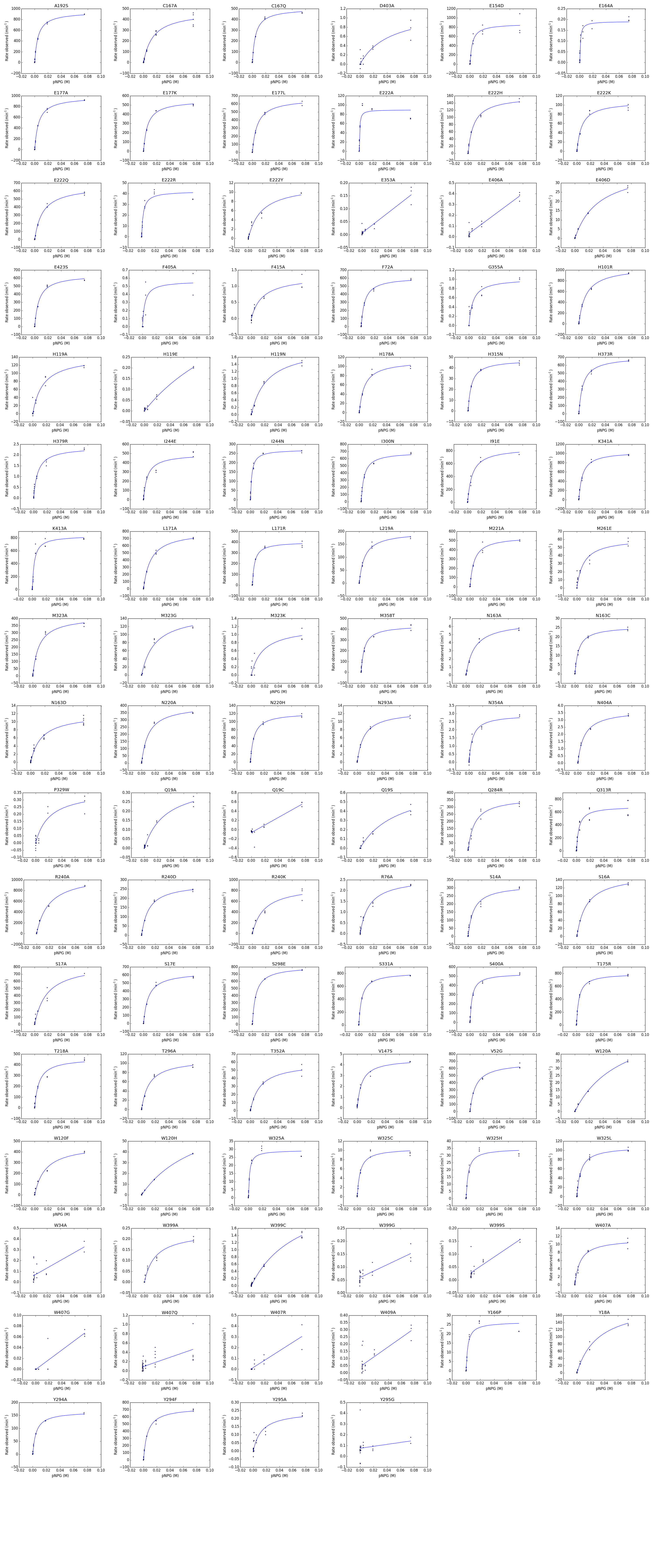

Supplement: S3 Fig — For each mutant, 8 observed rates (in triplicate) were fit to the Michaelis-Menten equation using SciPy and plots were generated using Matplotlib. Plots were used to visually confirm statistical analysis of the fits. (PNG) [file pone.0147596.s004.png]

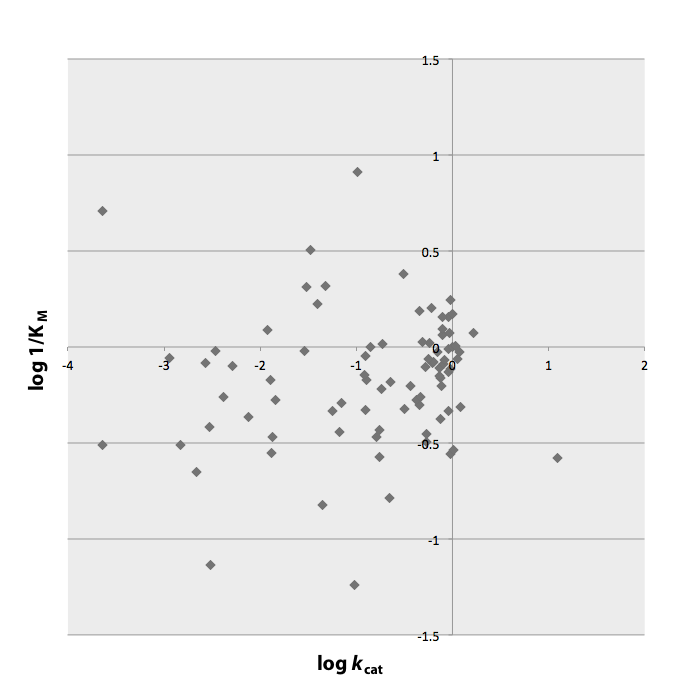

Supplement: S4 Fig — (TIFF) [file pone.0147596.s005.tiff]
