# Supplementary material for: Kinetic Characterization of 100 Glycoside Hydrolase Mutants Enables the Discovery of Structural Features Correlated with Kinetic Constants
Source: PLoS One. 2016 Jan 27;11(1):e0147596. doi: 10.1371/journal.pone.0147596 (PMC4729467; doi:10.1371/journal.pone.0147596)
Supplement: S1 Table — Included are columns (1) the mutation (2) protein yield as assessed by absorbance at 280 nm (3, 4, 5, 6) kinetic constants and nonlinear regression analysis for each of kcat, KM, KI, and kcat /KM. (DOCX) [file pone.0147596.s006.docx]

| **Mutant** | **Protein yield** | **K_M_** | ***k*_cat_** | **K_I_** | ***k*_cat_/K_M_** |
| --- | --- | --- | --- | --- | --- |
|  | **mg/mL** | **mM** | **min^-1^** | **mM** | **M^-1^min^-1^** |
| BglB | 1.2 | 5.00 ± 0.2 | 880 ± 10 |  | 176,000 ± 8000 |
| S14A | 0.6 | 8.25 ± 1.02 | 320 ± 11 |  | 38,823 ± 4,972 |
| S16A | 0.83 | 14.01 ± 0.40 | 154 ± 1 |  | 10,997 ± 331 |
| S17E | 1.01 | 7.32 ± 0.38 | 641 ± 9 |  | 87,596 ± 4,719 |
| S17A | 0.65 | 18.45 ± 3.72 | 848 ± 76 |  | 45,978 ± 10,135 |
| Y18A | 0.17 | 31.55 ± 3.61 | 197 ± 9 |  | 6,230 ± 773 |
| Q19A | 0.26 |  |  |  | 11 ± 3 |
| Q19C | 0.4 |  |  |  | < 10 |
| Q19S | 0.43 |  |  |  | 13 ± 3 |
| W34A | ND |  |  |  |  |
| V52G | 0.97 | 8.25 ± 0.54 | 687 ± 13 |  | 83,371 ± 5,707 |
| F72A | 0.44 | 5.47 ± 0.28 | 613 ± 8 |  | 112,224 ± 6,000 |
| R76A | ND |  |  |  |  |
| I91E | 0.49 | 6.71 ± 0.79 | 846 ± 35 |  | 126,071 ± 15,714 |
| H101R | 1.03 | 10.62 ± 0.53 | 1059 ± 16 |  | 99,708 ± 5,225 |
| H119A | 1.21 | 15.10 ± 3.36 | 143 ± 11 |  | 9,483 ± 2,222 |
| H119E | ND |  |  |  |  |
| H119N | 1.02 | 23.22 ± 2.20 | 2 ± <1 |  | 82 ± 8 |
| W120A | 0.16 |  |  |  | < 10 |
| W120F | 0.78 | 16.08 ± 2.07 | 472 ± 21 |  | 29,334 ± 3,980 |
| W120H | 1 | 89.18 ± 4.31 | 84 ± 2 |  | 943 ± 53 |
| V147S | 0.23 | 6.45 ± 0.62 | 5 ± <1 |  | 706 ± 70 |
| E154D | 1.42 | 3.46 ± 0.76 | 878 ± 47 |  | 254,004 ± 57,175 |
| N163A | 0.74 | 11.95 ± 0.91 | 7 ± <1 |  | 558 ± 44 |
| N163C | 1.1 | 5.42 ± 0.32 | 26 ± <1 |  | 4,766 ± 291 |
| N163D | 1.05 | 15.19 ± 1.41 | 12 ± <1 |  | 789 ± 77 |
| E164A | 0.42 | 1.01 ± 0.17 | < 1 |  | 190 ± 33 |
| Y166P | 0.18 | 2.50 ± 0.45 | 27 ± 1 | 94.95 ± 10.18 | 10,596 ± 1,981 |
| C167A | 0.48 | 14.56 ± 1.27 | 479 ± 14 |  | 32,884 ± 3,026 |
| C167Q | 0.94 | 4.92 ± 0.19 | 504 ± 6 | 590.71 ± 86.56 | 102,415 ± 4,149 |
| L171A | 0.38 | 11.09 ± 0.42 | 807 ± 9 |  | 72,719 ± 2,851 |
| L171R | 1.06 | 3.36 ± 0.23 | 403 ± 7 |  | 120,146 ± 8,506 |
| T175R | 0.86 | 3.59 ± 0.15 | 801 ± 8 |  | 223,033 ± 9,663 |
| E177A | 0.96 | 5.98 ± 0.22 | 986 ± 10 |  | 164,804 ± 6,408 |
| E177K | 0.95 | 6.19 ± 0.30 | 555 ± 7 | 362.94 ± 36.97 | 89,609 ± 4,493 |
| E177L | 0.77 | 7.48 ± 0.36 | 670 ± 10 |  | 89,478 ± 4,555 |
| H178A | 0.25 | 7.67 ± 0.73 | 113 ± 3 | 173.34 ± 42.79 | 14,697 ± 1,463 |
| A192S | 1.17 | 5.09 ± 0.18 | 946 ± 10 |  | 185,848 ± 6,994 |
| T218A | 0.98 | 6.51 ± 0.94 | 464 ± 18 |  | 71,280 ± 10,669 |
| L219A | 0.47 | 7.87 ± 0.60 | 199 ± 5 |  | 25,262 ± 2,010 |
| N220A | 0.61 | 10.27 ± 0.68 | 405 ± 8 |  | 39,425 ± 2,745 |
| N220H | 1.12 | 5.14 ± 0.21 | 123 ± 1 |  | 23,874 ± 1,031 |
| M221A | 0.73 | 6.25 ± 0.60 | 547 ± 15 |  | 87,554 ± 8,701 |
| E222A | 0.29 | 0.63 ± 0.15 | 90 ± 4 | 95.24 ± 13.70 | 143,604 ± 36,130 |
| E222H | 0.7 | 8.54 ± 0.53 | 160 ± 3 |  | 18,695 ± 1,212 |
| E222K | 0.5 | 7.22 ± 0.75 | 108 ± 3 |  | 14,955 ± 1,618 |
| E222Q | 1.3 | 12.16 ± 0.65 | 668 ± 11 |  | 54,923 ± 3,084 |
| E222R | 0.15 | 2.48 ± 0.44 | 42 ± 2 |  | 17,098 ± 3,148 |
| E222Y | 0.7 | 18.43 ± 3.14 | 12 ± 1 |  | 636 ± 116 |
| R240A | 1.11 | 19.46 ± 1.17 | 11011 ± 258 |  | 565,763 ± 36,384 |
| R240D | 0.8 | 10.82 ± 0.47 | 282 ± 4 |  | 26,093 ± 1,196 |
| R240K | 1.4 | 17.67 ± 3.32 | 898 ± 59 |  | 50,829 ± 10,102 |
| I244E | 0.6 | 5.97 ± 1.04 | 497 ± 23 |  | 83,137 ± 14,963 |
| I244N | 0.21 | 2.15 ± 0.13 | 271 ± 4 |  | 126,176 ± 7,795 |
| M261E | 0.11 |  |  |  | 702 ± 73 |
| Q284R | 0.52 | 9.68 ± 1.35 | 370 ± 15 |  | 38,182 ± 5,550 |
| N293A | 0.68 | 9.67 ± 0.44 | 13 ± 0 |  | 1,313 ± 63 |
| Y294A | 0.59 | 4.98 ± 0.17 | 166 ± 2 |  | 33,260 ± 1,180 |
| Y294F | 0.73 | 5.99 ± 0.32 | 735 ± 11 |  | 122,751 ± 6,883 |
| Y295A | 0.69 |  |  |  | < 10 |
| Y295G | 0.77 |  |  |  | < 10 |
| T296A | 0.39 | 11.05 ± 0.77 | 109 ± 2 | 142.75 ± 28.67 | 9,904 ± 722 |
| S298E | 1.1 | 5.28 ± 0.05 | 809 ± 2 |  | 153,264 ± 1,391 |
| I300N | 1.51 | 4.48 ± 0.32 | 693 ± 13 |  | 154,732 ± 11,520 |
| Q313R | 1.07 | 3.58 ± 0.51 | 689 ± 24 |  | 192,373 ± 28,109 |
| H315N | ND |  |  |  |  |
| M323A | 0.35 | 9.34 ± 0.80 | 416 ± 11 | 126.29 ± 25.47 | 44,477 ± 3,991 |
| M323G | 0.88 | 19.21 ± 2.91 | 154 ± 9 |  | 7,998 ± 1,302 |
| M323K | ND |  |  |  |  |
| W325A | 0.26 | 1.61 ± 0.23 | 29 ± 1 | 171.02 ± 19.85 | 18,243 ± 2,607 |
| W325C | 0.22 | 4.18 ± 0.53 | 10 ± <1 | 159.19 ± 34.38 | 2,503 ± 327 |
| W325H | 1.12 | 3.08 ± 0.43 | 35 ± 1 | 143.45 ± 25.04 | 11,358 ± 1,645 |
| W325L | 1.08 | 5.74 ± 0.35 | 109 ± 2 |  | 18,909 ± 1,198 |
| P329W | ND |  |  |  |  |
| S331A | 0.89 | 4.34 ± 0.11 | 817 ± 5 |  | 188,306 ± 5,055 |
| K341A | 0.92 | 5.46 ± 0.33 | 1046 ± 17 |  | 191,689 ± 12,041 |
| T352A | 0.7 | 14.26 ± 1.76 | 60 ± 2 |  | 4,174 ± 541 |
| E353A | 0.56 |  |  |  | < 10 |
| N354A | 0.34 | 5.38 ± 0.67 | 3 ± <1 |  | 547 ± 70 |
| G355A | ND |  |  |  |  |
| M358T | 0.62 | 4.83 ± 0.48 | 436 ± 11 |  | 90,241 ± 9,225 |
| H373R | 1.19 | 6.31 ± 0.30 | 707 ± 9 |  | 112,169 ± 5,512 |
| H379R | 0.14 | 6.24 ± 0.84 | 2 ± <1 |  | 380 ± 53 |
| W399A | 0.96 | 16.65 ± 2.52 | < 1 |  | 14 ± 2 |
| W399C | 0.93 | 70.33 ± 5.89 | 3 ± <1 |  | 39 ± 4 |
| W399G | 1.27 |  |  |  | < 10 |
| W399S | 1.5 |  |  |  | < 10 |
| S400A | 0.41 | 3.22 ± 0.22 | 531 ± 9 |  | 164,795 ± 11,833 |
| D403A | ND |  |  |  |  |
| N404A | 1.41 | 9.42 ± 0.42 | 4 ± <1 |  | 393 ± 18 |
| F405A | ND |  |  |  |  |
| E406A | 0.57 |  |  |  | < 10 |
| E406D | 0.27 | 34.13 ± 2.57 | 39 ± 1 |  | 1,146 ± 94 |
| W407A | ND |  |  |  |  |
| W407G | ND |  |  |  |  |
| W407Q | ND |  |  |  |  |
| W407R | ND |  |  |  |  |
| W409A | ND |  |  |  |  |
| K413A | 1.11 | 2.92 ± 0.48 | 835 ± 33 |  | 285,858 ± 48,589 |
| F415A | 0.53 | 16.63 ± 4.00 | 1 ± <1 |  | 80 ± 20 |
| E423S | 1.08 | 6.60 ± 0.42 | 646 ± 12 | 317.35 ± 65.22 | 97,777 ± 6,431 |

**S1 Table: Kinetic constants for 100 computationally-designed BglB mutants.** Included are columns (1) the mutation (2) protein yield as assessed by absorbance at 280 nm, where ND indicates that the protein did not express above our limit of detection (3, 4, 5, 6) kinetic constants and nonlinear regression analysis for each of *k*_cat_, K_M_, KI, and *k*_cat_ /K_M_.
