# Supplementary material for: Kinetic Characterization of 100 Glycoside Hydrolase Mutants Enables the Discovery of Structural Features Correlated with Kinetic Constants
Source: PLoS One. 2016 Jan 27;11(1):e0147596. doi: 10.1371/journal.pone.0147596 (PMC4729467; doi:10.1371/journal.pone.0147596)
Supplement: S2 Table — PCC and SRC values for each individual structural feature, given by Rosetta short name. For explanation of each short name, see reference [26]. (DOCX) [file pone.0147596.s007.docx]

| **Feature** | **PCC (Kcat/K_M_）** | **SRC (Kcat/K_M_）** | **PCC (1/K_M_)** | **SRC (1/K_M_)** | **PCC (K_cat_)** | **SRC (K_cat_)** |
| --- | --- | --- | --- | --- | --- | --- |
| all_cst | 0.182 | 0.116 | 0.039 | 0.071 | 0.140 | 0.120 |
| fa_rep | 0.289 | 0.268 | 0.253 | 0.155 | 0.064 | 0.084 |
| hbond_sc | -0.352 | -0.352 | -0.248 | -0.297 | -0.309 | -0.266 |
| SR_1_all_cst | -0.061 | -0.039 | -0.148 | -0.114 | -0.163 | -0.119 |
| SR_1_burunsat_pm | 0.000 | 0.000 | 0.000 | 0.000 | 0.000 | 0.000 |
| SR_1_fa_rep | 0.195 | 0.180 | 0.193 | 0.309 | 0.155 | 0.042 |
| SR_1_hbond_pm | 0.000 | 0.000 | 0.000 | 0.000 | 0.000 | 0.000 |
| SR_1_hbond_sc | 0.000 | 0.000 | 0.000 | 0.000 | 0.000 | 0.000 |
| SR_1_nlpstat_pm | 0.142 | 0.087 | 0.263 | 0.317 | 0.007 | 0.055 |
| SR_1_pstat_pm | 0.164 | 0.096 | 0.050 | 0.064 | -0.131 | -0.069 |
| SR_1_total_score | -0.078 | 0.093 | -0.092 | -0.050 | -0.081 | 0.024 |
| SR_2_all_cst | 0.039 | -0.064 | -0.089 | -0.059 | -0.005 | -0.096 |
| SR_2_burunsat_pm | 0.000 | 0.000 | 0.000 | 0.000 | 0.000 | 0.000 |
| SR_2_fa_rep | 0.074 | 0.168 | 0.258 | 0.168 | 0.011 | 0.014 |
| SR_2_hbond_pm | 0.087 | 0.092 | 0.162 | 0.191 | 0.149 | 0.116 |
| SR_2_hbond_sc | -0.149 | -0.235 | -0.142 | -0.229 | -0.126 | -0.138 |
| SR_2_nlpstat_pm | 0.168 | 0.142 | 0.031 | 0.038 | 0.080 | 0.092 |
| SR_2_pstat_pm | 0.102 | 0.070 | -0.042 | -0.017 | -0.023 | 0.013 |
| SR_2_total_score | 0.071 | 0.002 | -0.078 | -0.079 | -0.055 | -0.134 |
| SR_3_all_cst | -0.061 | -0.039 | -0.148 | -0.114 | -0.163 | -0.119 |
| SR_3_burunsat_pm | 0.000 | 0.000 | 0.000 | 0.000 | 0.000 | 0.000 |
| SR_3_fa_rep | 0.195 | 0.180 | 0.193 | 0.309 | 0.155 | 0.042 |
| SR_3_hbond_pm | 0.000 | 0.000 | 0.000 | 0.000 | 0.000 | 0.000 |
| SR_3_hbond_sc | 0.000 | 0.000 | 0.000 | 0.000 | 0.000 | 0.000 |
| SR_3_nlpstat_pm | 0.142 | 0.087 | 0.263 | 0.317 | 0.007 | 0.055 |
| SR_3_pstat_pm | 0.164 | 0.096 | 0.050 | 0.064 | -0.131 | -0.069 |
| SR_3_total_score | -0.078 | 0.093 | -0.092 | -0.050 | -0.081 | 0.024 |
| SR_4_all_cst | 0.000 | 0.000 | 0.000 | 0.000 | 0.000 | 0.000 |
| SR_4_burunsat_pm | 0.123 | 0.135 | 0.089 | 0.078 | 0.067 | 0.138 |
| SR_4_fa_rep | -0.118 | -0.024 | -0.235 | -0.212 | -0.153 | -0.103 |
| SR_4_hbond_pm | -0.197 | -0.206 | 0.046 | 0.069 | -0.211 | -0.182 |
| SR_4_hbond_sc | 0.299 | 0.092 | -0.009 | -0.063 | 0.058 | 0.061 |
| SR_4_nlpstat_pm | 0.067 | 0.174 | 0.160 | 0.244 | -0.007 | -0.015 |
| SR_4_pstat_pm | 0.040 | 0.098 | 0.105 | 0.152 | -0.025 | -0.072 |
| SR_4_total_score | 0.096 | 0.045 | 0.193 | -0.121 | -0.181 | -0.076 |
| SR_5_all_cst | 0.128 | 0.074 | -0.091 | -0.048 | -0.062 | -0.082 |
| SR_5_burunsat_pm | 0.055 | 0.052 | 0.061 | -0.031 | 0.136 | 0.099 |
| SR_5_dsasa_1_2 | 0.235 | 0.189 | 0.037 | 0.147 | 0.215 | 0.201 |
| SR_5_fa_rep | -0.012 | -0.064 | 0.165 | -0.019 | 0.058 | -0.030 |
| SR_5_hbond_pm | 0.514 | 0.410 | 0.267 | 0.334 | 0.267 | 0.146 |
| SR_5_hbond_sc | -0.524 | -0.447 | -0.266 | -0.341 | -0.273 | -0.194 |
| SR_5_interf_E_1_2 | -0.461 | -0.469 | -0.237 | -0.231 | -0.266 | -0.278 |
| SR_5_total_score | -0.462 | -0.471 | -0.237 | -0.230 | -0.267 | -0.279 |
| tot_burunsat_pm | -0.078 | -0.101 | -0.036 | -0.066 | 0.154 | 0.056 |
| tot_hbond_pm | 0.419 | 0.396 | 0.291 | 0.380 | 0.315 | 0.323 |
| tot_NLconts_pm | 0.570 | 0.548 | 0.246 | 0.238 | 0.432 | 0.421 |
| tot_nlpstat_pm | 0.334 | 0.313 | 0.223 | 0.164 | 0.277 | 0.277 |
| tot_nlsurfaceE_pm | -0.285 | -0.284 | -0.193 | -0.276 | -0.176 | -0.111 |
| tot_pstat_pm | 0.306 | 0.209 | 0.118 | -0.009 | 0.221 | 0.161 |
| tot_seq_recovery | 0.000 | 0.000 | 0.000 | 0.000 | 0.000 | 0.000 |
| tot_total_charge | 0.051 | 0.053 | 0.199 | 0.153 | 0.103 | 0.123 |
| tot_total_neg_charges | -0.068 | -0.063 | -0.148 | -0.090 | -0.044 | -0.034 |
| tot_total_pos_charges | 0.010 | 0.014 | 0.177 | 0.209 | 0.133 | 0.129 |
| total_score | -0.340 | -0.396 | -0.059 | -0.014 | -0.320 | -0.362 |

**S2 Table. Correlations between individual structural features and each of *k*_cat_, K_M_, and *k*_cat_/K_M_.** PCC and SRC values for each individual structural feature, given by Rosetta short name. For explanation of each short name, see main text.
