# Supplementary material for: Kinetic Characterization of 100 Glycoside Hydrolase Mutants Enables the Discovery of Structural Features Correlated with Kinetic Constants
Source: PLoS One. 2016 Jan 27;11(1):e0147596. doi: 10.1371/journal.pone.0147596 (PMC4729467; doi:10.1371/journal.pone.0147596)
Supplement: S3 Table — A multiple sequence alignment of 1,554 family 1 glycoside hydrolases from the Pfam database aligned to the BglB wild type sequence was used for this analysis. Column 1 is the relative kcat/KM compared to wild type on a log scale. Column two gives the position and native BglB residue at that position. Column three is the percentage of the 1,554 aligned sequences that have the same residue as BglB. (DOCX) [file pone.0147596.s008.docx]

| ***k*_cat_/K_M_** | **Mutant** | **Conservation (%)** |
| --- | --- | --- |
| 0.52 | R240A | 13 |
| 0.22 | K413A | 27 |
| 0.05 | K341A | 16 |
| 0.04 | S331A | 60 |
| -0.02 | S400A | 41 |
| -0.02 | E177A | 3 |
| -0.08 | E222A | 7 |
| -0.19 | F72A | 44 |
| -0.29 | M221A | 3 |
| -0.37 | L171A | 11 |
| -0.38 | T218A | 17 |
| -0.57 | S17A | 52 |
| -0.59 | M323A | 10 |
| -0.64 | N220A | 19 |
| -0.65 | S14A | 61 |
| -0.71 | Y294A | 49 |
| -0.72 | C167A | 11 |
| -0.83 | L219A | 22 |
| -0.97 | W325A | 70 |
| -1.07 | H178A | 7 |
| -1.19 | S16A | 48 |
| -1.24 | T296A | 14 |
| -1.26 | H119A | 96 |
| -1.44 | Y18A | 25 |
| -1.62 | T352A | 40 |
| -2.06 | W407A | 44 |
| -2.12 | N293A | 50 |
| -2.48 | N163A | 96 |
| -2.49 | N354A | 86 |
| -2.64 | N404A | 34 |
| -2.94 | E164A | 100 |
| -2.95 | R76A | 96 |
| -3.01 | G355A | 96 |
| -3.34 | F415A | 47 |
| -4.16 | W399A | 96 |
| -4.54 | E406A | 47 |
| -4.76 | Q19A | 95 |
| -4.93 | E353A | 100 |
| ND | Y295A | 100 |
| ND | D403A | 98 |
| ND | W34A | 40 |
| ND | W120A | 34 |
| ND | F405A | 26 |
| ND | W409A | 10 |
| 0.00 | BglB | NA |

**S3 Table. Conservation analysis of BglB active site residues.** A multiple sequence alignment of 1,554 family 1 glycoside hydrolases from the Pfam database aligned to the BglB wild type sequence was used for this analysis. Column 1 is the relative *k*_cat_/K_M_ compared to wild type on a log scale. Column two gives the position and native BglB residue at that position. Column three is the percentage of the 1,554 aligned sequences that have the same residue as BglB.
