# Supplementary material for: Kinetic Characterization of 100 Glycoside Hydrolase Mutants Enables the Discovery of Structural Features Correlated with Kinetic Constants
Source: PLoS One. 2016 Jan 27;11(1):e0147596. doi: 10.1371/journal.pone.0147596 (PMC4729467; doi:10.1371/journal.pone.0147596)

**S2 Text. Prediction and feature selection via Elastic net**. A regularized linear regression model, Elastic Net (EN), was chosen to fit the dataset of the kinetic constants, each constant fitted independently. Comparing to ordinary least squares regression, an EN model is able to make a prediction and select the most informative feature set simultaneously as and penalties are applied to the regression weights. The weight of each structural feature is estimated as

Where:

: the intercept;

: the weight of structural feature i in the regression model;

p: the number of structural features generated by the BglB model;

: the kinetic constant (the dependent variable to be predicted);

: structural features generated by the BglB model (the independent variables);

,: parameters tuning the constraints on the weights.

Since the structural feature were measured in different ranges and units, we first normalized all the features to be zero-centered with variance being one by subtracting the mean and dividing by the variance of the feature value. All the features are on the same scale to compare their contribution to the kinetic constants after the normalization. The tuning parameters, are determined one by one via stratified 10-fold cross validation by searching a grid of and. Each round of cross validation generated a linear regression model. In order to build a more generalized model, cross validation was run 1,000 times with a different part of the dataset each time. The final prediction of a mutant’s kinetic constant was an average of all the predictions during the 1,000 rounds of training. The average number of non-zero weights when predicting *k*_cat_/K_M_, *k*_cat_ and K_M_ were 9, 8 and 10 respectively. The top features were chosen and listed in table 1 with their averaged weights among all the models (9 for *k*_cat_/K_M_, 8 for *k*_cat_, 10 for K_M_)**.** Stratified 10-fold cross validation was implemented to validate the EN model. Specifically, all the mutants were first ranked according to the experimentally-measured value of the kinetic constant to be predicted and every 10 adjacent datapoints were randomly marked with an index using integers from 1 to 10 without duplication. Finally, all the datapoints with the same index were grouped together, resulting in ten folds. Since the datapoints in each folds comes from different level of the dataset, this guarantees every fold is a good representative of the dataset. In order to build a robust prediction model, the cross validation was run 1,000 times, the dataset split into training set and testing set differently each time.

Each bootstrap set is constructed by uniformly sampling from the original database, with a coverage of 87%, which is calculated as $1-({1-1/n)}^{2n}$. The following figure shows the prediction of the left-out subsets during bootstrapping.


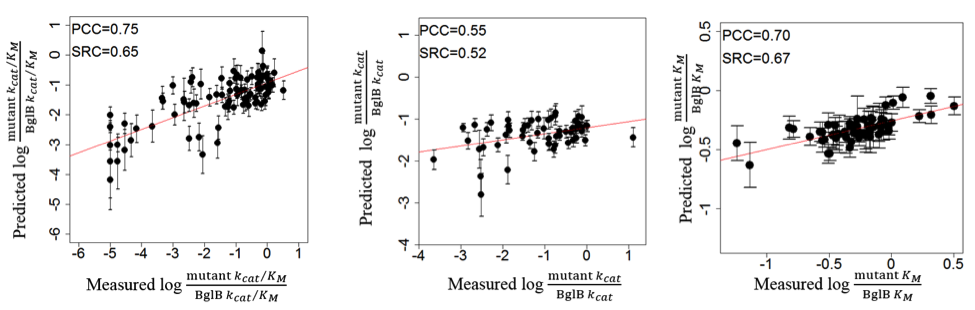

Supplement: S2 Text — (DOCX) [file pone.0147596.s010.docx]
